# Effects of Me–Solvent Interactions on the Structure and Infrared Spectra of MeTFSI (Me = Li, Na) Solutions in Carbonate Solvents—A Test of the GFN2-xTB Approach in Molecular Dynamics Simulations

**DOI:** 10.3390/molecules28186736

**Published:** 2023-09-21

**Authors:** Piotr Wróbel, Andrzej Eilmes

**Affiliations:** Faculty of Chemistry, Jagiellonian University, Gronostajowa 2, 30-387 Kraków, Poland

**Keywords:** ethylene carbonate, liquid electrolytes, salt–solvent interaction, IR spectra, molecular dynamics, GFN2-xTB method

## Abstract

We investigated the performance of the computationally effective GFN2-xTB approach in molecular dynamics (MD) simulations of liquid electrolytes for lithium/sodium batteries. The studied systems were LiTFSI and NaTFSI solutions in ethylene carbonate or fluoroethylene carbonate and the neat solvents. We focused on the structure of the electrolytes and on the manifestations of ion–solvent interactions in the vibrational spectra. The IR spectra were calculated from MD trajectories as Fourier transforms of the dipole moment. The results were compared to the data obtained from ab initio MD. The spectral shifts of the carbonyl stretching mode calculated from the GFN2-xTB simulations were in satisfactory agreement with the ab initio MD data and the experimental results for similar systems. The performance in the region of molecular ring vibrations was significantly worse. We also found some differences in structural data, suggesting that the GFN2-xTB overestimates interactions of Me ions with TFSI anions and Na^+^ binding to solvent molecules. We conclude that the GFN2-xTB method is an alternative worth considering for MD simulations of liquids, but it requires testing of its applicability for new systems.

## 1. Introduction

Rechargeable energy storage devices play an important role in the modern world, addressing the issues of changeable power supply from environmentally friendly sources, such as photovoltaics or wind farms. In everyday life, metal-ion batteries find numerous applications, from portable electronics to electric vehicles. From these devices, commercially, the most successful are the lithium-ion batteries (LIBs) since their introduction to the market in the 1990s [1,2,3]. With shrinking natural resources and increasing economic tensions around the world, concerns about stable supplies of lithium salts stimulate interest in alternative chemistries, e.g., sodium or magnesium batteries [3,4,5,6,7,8,9,10].

A typical electrolyte used in commercial LIBs is the solution of a lithium salt in an organic liquid (such as linear or cyclic carbonate) [11]. A commonly used solvent is ethylene carbonate (EC). The electrolyte properties and safety of use can be improved by additives. Among them are fluorinated solvents, including fluorinated derivatives of ethylene carbonate, decreasing the flammability, contributing to the formation of the solid-electrolyte interphase layer, and improving the electrochemical stability and overall performance of the device [12,13,14]. Fluorinated additives have been tested for lithium and sodium batteries [12,13,14,15,16,17,18,19].

Vibrational spectroscopy (Raman or infrared, IR) is quite commonly used to study the solvent–salt interactions and the structure of the electrolytes [17,20,21,22,23,24,25], owing to the sensitivity of vibrational frequencies to the environment of the oscillating group, and to complexation by metal ions. Such experiments are supported by quantum chemical (QC) methods, yielding information on the structure of the solvation shell, strength of ion–solvent interactions, and vibrational frequencies [22,26,27,28,29,30,31]. QC calculations are typically performed in a vacuum, whereas the interactions in the real electrolyte occur in the condensed phase. The presence of the solvent and its effect on binding energies and vibrational frequencies can be accounted for by applying a continuous solvent model [30,31,32]. The bulk solution can be effectively modeled using classical (force-field-based) molecular dynamics (MD) simulations. These are, however, rather unsuitable for investigations of vibrational spectra, but they deliver valuable information on the structure and dynamical properties of the electrolyte [33,34,35,36,37,38].

Vibrational spectra can be obtained from ab initio molecular dynamics (AIMD) simulations using Fourier transforms (FTs) of the autocorrelation function of the dipole moment (IR spectrum) or the polarizability (Raman spectrum) of the system [39]. Simulations of this kind have been reported for several systems, including molecular liquids [40] and studies on ion–solvent interactions in liquid electrolytes [38,41,42]. In our previous work on metal ion interactions with cyclic carbonates [38], we used AIMD simulations applying density functional theory (DFT) and the PBE functional to study electrolytes with lithium and sodium bis(trifluoromethylsulfonyl) imide salts (LiTFSI and NaTFSI) dissolved in EC and its fluorinated derivative. The calculated IR spectra reproduced the shifts in the vibrational modes of the solvent induced by interactions with salt cations. Through a detailed analysis of the local structure of the electrolyte, we were able to correlate the frequencies of local oscillations to the coordination environments of carbonate molecules.

AIMD simulations are a valuable tool in physical chemistry, yet their applications are still limited by the high computational cost of the ab initio methods, despite the increasing power of computer resources. Therefore, less computationally demanding approaches are considered, such as semi-empirical methods. Among these is the density-functional-based tight binding (DFTB) approach [43,44]. DFTB has been already used in modeling the vibrational spectra of liquids from MD simulations [45,46]. The DFTB methodology can increase the speed of computations, but it requires parameterization. The parameters of the repulsive potential are needed for each combination of chemical elements present in the system. Several general-use parameterizations for biological system or material modeling are available, as well as those tailored for a specific compound, but when the new studied system contains elements not covered by the existing parameter set, new parameters have to be developed. This is a serious disadvantage of DFTB. In particular, none of the publicly available parameterizations contains Li, limiting the applicability of DFTB to lithium electrolytes. Therefore, in our test of the DFTB approach performed in [38], we were able to simulate only the neat solvents and the NaTFSI solution. The DFTB-based MD yielded a much worse reproduction of spectral IR shifts in the electrolyte [38].

A possible alleviation of the problems with the limited availability of DFTB parameters comes from a general and broadly parameterized version of DFTB3, called GFN-xTB [47,48]. In our recent study on the hydrogen bonding and IR spectra of ionic liquid/water mixtures [49], we tested two variants of this approach: GFN1-xTB and GFN2-xTB. The performance of the GFN2-xTB method, compared to DFT/PBE AIMD, was quite reasonable for neat liquids; the description of mixtures was less satisfactory [49]. Encouraged by the results of [49], we decided to test the GFN2-xTB approach on the MeTFSI solutions in carbonates, investigated earlier via AIMD and DFTB MD [38].

To this end, we performed MD simulations for LiTFSI and NaTFSI solutions in EC and fluoroethylene carbonate (F1EC), using the GFN2-xTB method to solve the electron structure. In the following sections, we present the structure of the electrolytes (ion coordination), IR spectra calculated from the MD trajectories, and an analysis of local oscillations based on FTs of selected interatomic distances. The results are discussed with respect to the AIMD simulations [38], which provided a good reproduction of the experimentally observed spectral shifts and therefore served as a reference.

## 2. Results

In this section, we present the results of MD simulations using the GFN2-xTB approach, compared to the results of our previous study [38]. For brevity, in this text, we refer to the current calculations as xTB results, whereas the DFT simulations using the PBE functional and the DFTB/3ob-3-1 results from [38] are denoted as AIMD and DFTB data, respectively.

### 2.1. Structure of the Electrolytes

**Figure 1:**
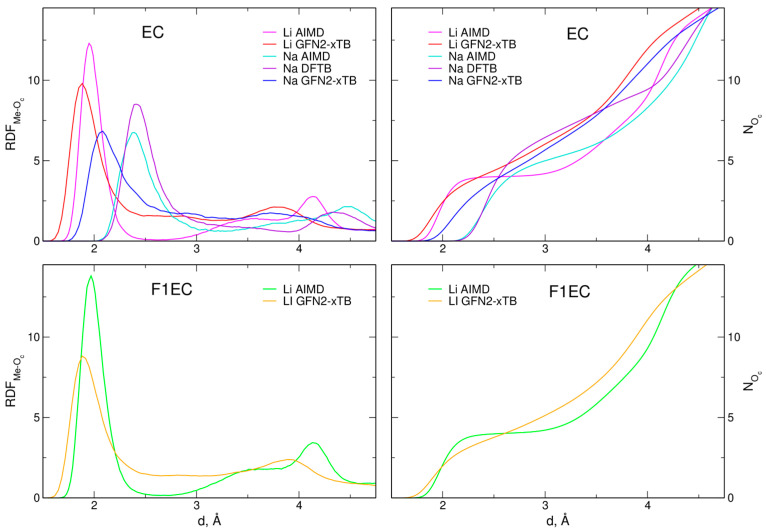
Radial distribution functions and integrated RDFs for Me–O_c_ atom pairs calculated from the MD simulations of electrolytes. AIMD and DFTB data were obtained from [38].

To analyze the structure of the MeTFSI solutions, we computed the radial distribution functions (RDFs) between metal ions and oxygen atoms from the C=O group of the carbonate molecule (O_c_ atoms) or from TFSI anions (labeled O_T_). In Figure 1, we show the RDFs for Me–O_c_ atom pairs and the integrated RDFs (running coordination numbers, CNs), yielding the average CNs of Me cations. For easy comparison to the DFT-based AIMD and DFTB results, we also included the data from other approaches, as calculated in [38].

The first maximum in the Li–O_c_ RDFs in the xTB simulations appears at about 1.9 Å, regardless of the solvent (EC or F1EC). Its position is shifted to a distance slightly lower than that obtained in AIMD (1.95–1.97 Å). It is also noticeable that the maximum in the xTB results is wider, and, unlike the AIMD results, the RDFs do not decrease to zero at about 2.5 Å. The first maximum of the Na–O_c_ RDF is located at 2.1 Å. Although the Na–O_c_ distances are larger than those for Li–O_c_, this maximum in the xTB data is at the distance about 0.3 Å shorter than that calculated within AIMD via the DFTB methodology.

Like the RDFs, the coordination numbers for Li^+^ cations are also similar in both solvents. The integrated Li–O_c_ RDFs obtained in the xTB simulations do not exhibit the plateau observed between 2 and 3 Å in the AIMD results. Within the distance of 2.5 Å from the central metal ion, there are, on average, 4.4 and 3.75 O_c_ atoms from EC and F1EC molecules, respectively. These values are similar to the AIMD results (4.0 and 3.98, respectively). Owing to the larger radius of the Na^+^ ion, its CN in the NaTFSI–EC solution is larger: within the distance 3 Å, there are 5.65 and 5.0 O_c_ atoms in the xTB and AIMD trajectories, respectively. The CN = 6.45 obtained from the DFTB data was the largest. The main results related to the Me–O_c_ RDFs are summarized in Table 1.

**Figure 2:**
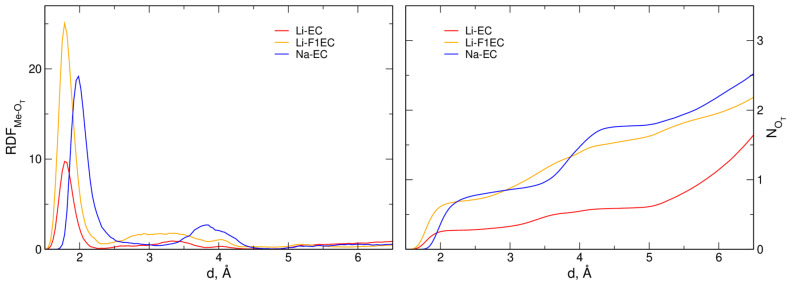
Radial distribution functions and integrated RDFs for Me–O_T_ atom pairs in GFN2-xTB MD simulations.

At a low salt concentration, there is practically no Me coordination to TFSI anions in AIMD simulations [38]. Accordingly, in Figure 2, we present the Me–O_T_ RDFs only for the xTB data; the results from both approaches are compared in Table 2. Similarly to the Li–O_c_ RDFs, the first maximum in the Li–O_T_ RDF is at the same distance, 1.78 Å, in EC and F1EC. The maximum for Na–O_T_ pairs appears at 1.99 Å. In all cases, the distance of the maximum is about 0.1 Å smaller than for Me–O_c_. The Me–O_T_ CNs, calculated at the same distances as for O_c_ atoms above, are 0.28, 0.71, and 0.85 for Li-EC, Li-F1EC, and Na-EC electrolytes, respectively.

### 2.2. IR Spectra

**Figure 3:**
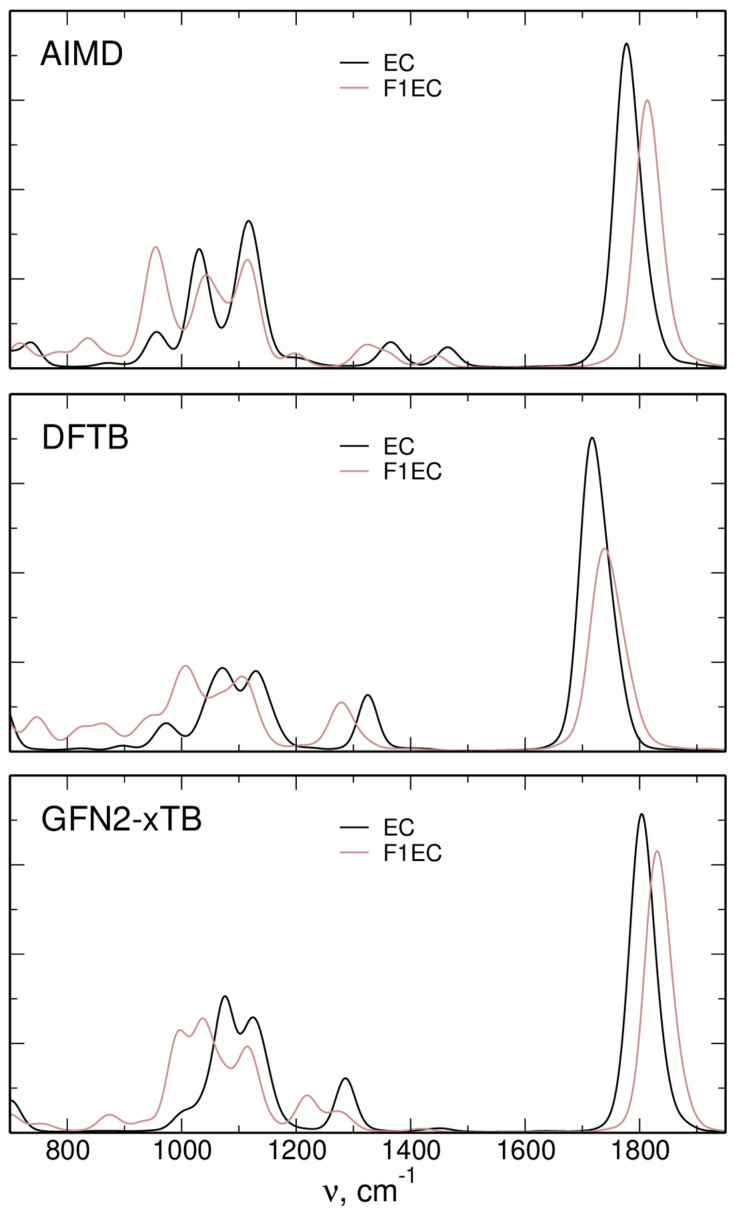
Simulated IR spectra of neat solvents. AIMD and DFTB data were obtained from [38].

In Figure 3, we compare the IR spectra obtained for neat EC and F1EC liquids from AIMD, DFTB [38], and xTB simulations. The frequencies of the C=O stretching vibration around 1800 cm^−1^ in xTB (1803 cm^−1^ for EC and 1831 cm^−1^ for F1EC) are higher than the AIMD values (1777 cm^−1^ and 1814 cm^−1^, respectively) but are in much better agreement with the latter than with the DFTB results. The difference between the F1EC and EC C=O frequencies reads as 37, 22, and 28 cm^−1^ in AIMD, DFTB, and xTB simulations, respectively, with the xTB result being between the values obtained in the two other approaches.

In the region of ring vibrations, the general features of the spectra are similar, with some changes in the frequencies and intensities. Overall, the calculated intensity pattern in the xTB data is closer to the DFTB result. The other vibration, interesting in the context of ion–carbonate interactions, is the ring-breathing mode below 1000 cm^−1^. Its position in the xTB spectra is shifted to a higher frequency compared to that obtained via the AIMD approach. In both cases, its intensity for F1EC is larger than that for EC, in accordance with the larger changes of the dipole moment during ring-breathing oscillation of an asymmetric F1EC molecule.

**Figure 4:**
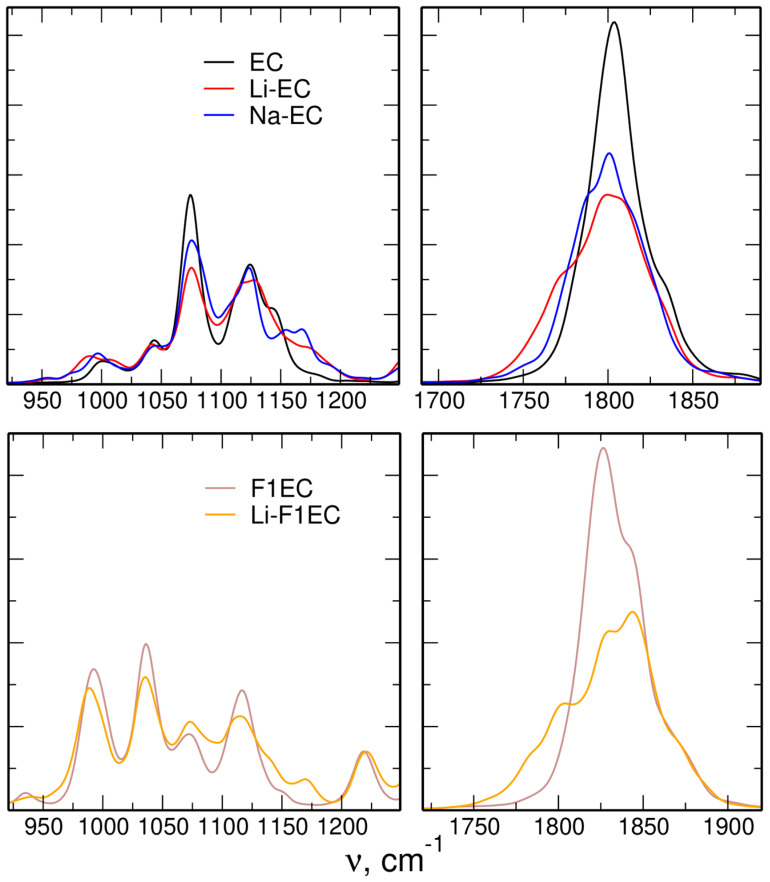
IR spectra of neat solvents and MeTFSI solutions calculated from the GFN2-xTB MD simulations.

The IR spectra calculated in this work for solvents and electrolytes within the GFN2-xTB approach are shown in Figure 4. In the region of the C=O mode, there is a visible red-shift of the intensity observed in the salt solutions. In the spectrum of the LiTFSI electrolyte in F1EC, an additional peak appears at the low-frequency side of the main band. In the spectra calculated for EC solutions, there is no additional maximum, but a shoulder appears instead. The down-shifts of these features are −27, −28, and −15 cm^−1^ for the Li-F1EC, Li-EC, and Na-EC electrolytes, respectively. The changes observed in the lower part of the spectra are less systematic, and there is no clear effect of Me–solvent interactions. In particular, there is no blue-shift of the ring-breathing mode, expected from a previous experiment [17]. We discuss this issue later in Section 3.

**Figure 5:**
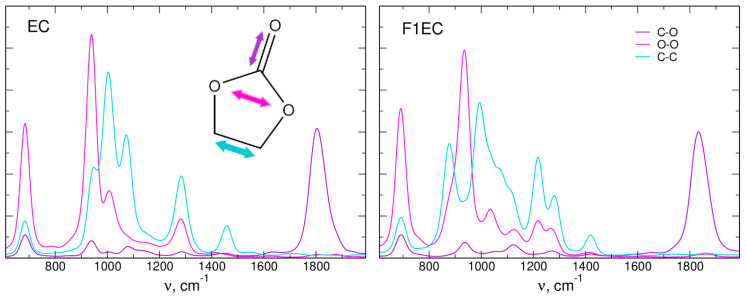
Power spectra obtained as FTs of interatomic distances of EC and F1EC molecules in GFN2-xTB simulations for neat solvents.

In order to obtain better insight into the origin of the IR bands observed in Figure 4, we calculated the FTs of selected geometrical parameters of carbonate molecules: lengths of C=O and C-C bonds and the distance between the two O atoms in the ring. The resulting power spectra are shown in Figure 5. As expected, the C=O stretching frequency corresponds to the IR band at 1800 cm^−1^. The oscillations of the O-O distance contribute to several modes, including vibrations at about 950 cm^−1^ and 1000 cm^−1^. The modes between 900 cm^−1^ and 1500 cm^−1^ also involve C-C stretching. The latter contribution is the most changed between EC and F1EC; this is not surprising, because the fluorination site is at one of the C atoms.

**Figure 6:**
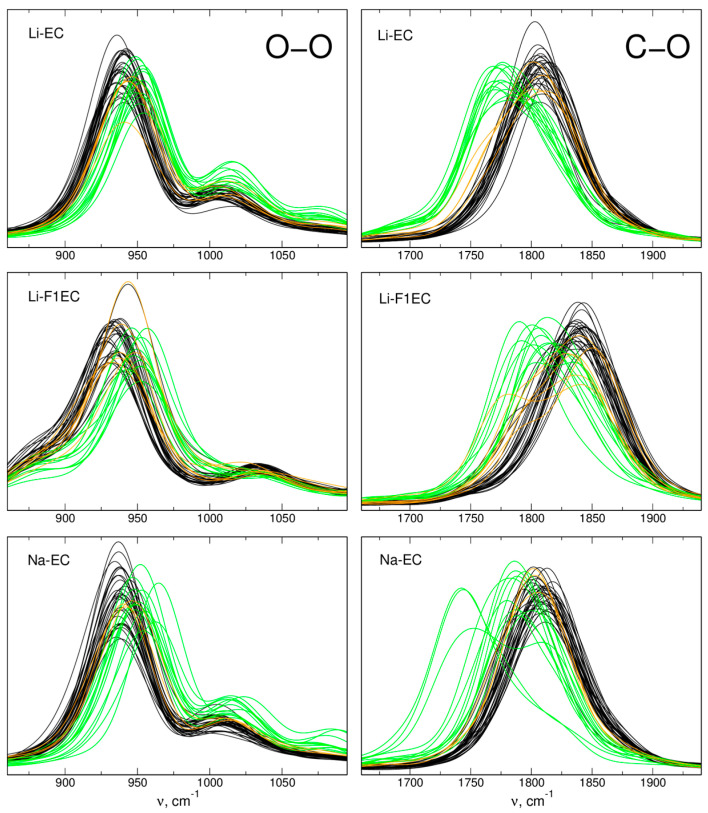
FTs of O-O distances and C=O bond lengths for individual solvent molecules in GFN2-xTB simulations. Free solvent molecules are black, molecules interacting with Me^+^ are green, and orange lines correspond to molecule interaction with a cation only in a part of the trajectory.

The FTs of interatomic distances are helpful in the analysis of the effect of the local environment of an oscillator on its vibrational frequency. Examples of such an analysis are displayed in Figure 6. Here, we obtained the FTs of selected distances (carbonyl C-O and ring O-O) for each solvent molecule in the sample and labeled the data according to the coordination of the molecule to the metal cation during the MD simulation. The molecules interacting with the ion for more than 25 ps are considered “coordinated” and shown in green; molecules coordinated for 10–25 ps are marked orange; and those interacting for less than 10 ps are considered “free” and shown in black.

As can be readily seen, interactions with Me^+^ shift the C=O oscillations to lower frequencies—the maxima of all green lines are red-shifted with respect to the average frequency of free solvent molecules. Ion complexation has an opposite effect on the O-O power spectrum. Although the changes are smaller, the interaction apparently shifts the oscillation to a higher frequency. The values of both kinds of shifts depend on the solvent and the ion, but the qualitative picture is the same regardless of the interacting species. The results shown in Figure 6 agree with the effect calculated in AIMD simulations [38], and the directions of the shifts are consistent with QC calculations for solvent–ion pairs [30] and the experimental vibrational spectra [17,24,25].

## 3. Discussion

The GFN2-xTB approach predicts lower Me–O_c_ distances in MD simulations than the DFT/PBE calculations. For Li^+^ cations, the difference is 0.05–0.07 Å and the xTB distances are similar to the results of classical MD [38]. Shortening of the metal–carbonate distance is particularly pronounced for Na^+^ interactions with EC, where it amounts to 0.3 Å, and it is larger than that calculated in classical MD simulations. It is also noticeable that the differences between the xTB and DFTB results, with respect to the AIMD data, are in opposite directions.

Wider maxima in the RFDs for Me–O_c_ atom pairs and the lack of a plateau in the integrated RDFs indicate that the solvation shell of the cation in xTB simulations is more diffuse in comparison to the quite compact shell obtained in AIMD. Therefore, the differences in the strength of ion–carbonate interactions for individual solvent molecules coordinating the ion will probably be larger.

The major difference between the GFN2-xTB and AIMD results is that the former predicted Me^+^ coordination to TFSI anions even at a 1 M salt concentration. The average number of coordinated O_T_ atoms is the smallest for LiTFSI solution in EC, and for the two other electrolytes, the Me–O_T_ CNs do not exceed 1, but in all cases, the Me–TFSI interactions are non-negligible. The results for LiTFSI solutions show that there is competition between Li^+^ interactions with the solvent and with the salt anions. The Li–O_c_ CN in F1EC is smaller than that in the EC-based electrolyte, what may be rationalized by weaker Li–carbonate interactions, as shown by the QC-calculated interaction energies [30]. Nevertheless, the total CNs of Me ions (that is, including both O_c_ and O_T_ atoms) are 4.68 and 4.46 for EC and F1EC solutions, respectively. Therefore, the total CNs are similar in both solvents, despite a larger difference in the Me–O_T_ CNs.

The results of the current simulations can also be compared to experimental structural information on LiTFSI solutions in EC [25]. The xTB-calculated Li–O_c_ distance of 1.9 Å is very similar to the experimentally determined 1.91 Å [25]; this agreement is even better than that for AIMD results. However, this is not the case for the Li–O_T_ distance: according to the xTB simulations, it is shorter than the Li–O_c_ distance, whereas the experimental result of 1.98 Å shows the opposite trend. The latter value is in agreement with the AIMD data for a concentrated electrolyte [38]. The experimental CN for Li cations at a low salt concentration is 3.8—lower than the xTB result of 4.7. Moreover, in dilute solutions, there are no TFSI anions in the first solvation shell, as determined from neutron diffraction data and Raman spectra [25]. The increased calculated Li–O_T_ CN is consistent with too-short Li–TFSI distances. We can conclude that the GFN2-xTB method used for MD simulations of MeTFSI solutions in carbonate solvents overestimates the strength of Me^+^ interactions with anions and the interactions of Na^+^ with the solvent.

The possible origin of differences between methods is the construction of the GFN2-xTB parameterization. It uses only element-specific parameters, and the repulsion potential for atom pairs is constructed based on element-wise parameters. The element-specific parameters were set in a way to provide the best overall reproduction of results over several benchmark sets. However, there is a possibility that these general parameters may give worse performance when applied to a specific pair interaction, e.g., between a cation and a polar group (Na^+^ and carbonyl). Likewise, the pair potential constructed from atomic parameters will not discriminate between different environments of atoms, e.g., between Me^+^ interaction with O_c_ or O_T_ atoms. Conversely, the DFTB method uses pair-specific parameters, allowing, in principle, for better adjustment to a given system, but at the cost of parameterization development being necessary for new classes of systems.

Turning our attention to the MD-simulated IR spectra, we can assume that the agreement with the xTB and AIMD results for neat EC and F1EC liquids is acceptable. Therefore, we focus on the analysis of interaction-induced spectral shifts, which are sensitive to more subtle effects of interactions in the solution. For the reader’s convenience, the main results are presented in Table 3.

In the region of the C=O stretching mode, the agreement between the two approaches is quite impressive. The local modes due to coordinated solvent molecules in the xTB spectra are not as sharply separated from the main band as in the AIMD data (shoulders rather than local peaks appear in the spectrum), but this can be attributed to a more diffuse solvation shell of Me cations. The shifts of −28 cm^−1^ for Li and −15 cm^−1^ for Na calculated in the xTB-based MD are similar to the values −33, −25, and −16 cm^−1^ obtained in [38] for Li-EC, Li-F1EC, and Na-EC electrolytes. These results are also in reasonable agreement with the experimental values: −30 and −36 cm^−1^ for LiTFSI and a smaller shift of −20 cm^−1^ for NaTFSI in linear carbonates [17,24].

The case of vibrations of the molecular ring is much less clear. From the experimental vibrational spectra [17,25], it is known that the interactions with metal ions result in a blue-shift of the ring-breathing mode of the EC or F1EC molecule. Such an effect was correctly reproduced in QC calculations [30] and in the AIMD spectra [38]. However, no systematic changes appear in the xTB-based IR spectra in the left-column panels of Figure 4, and for some bands, the shifts are in the direction opposite to the experimental trend. On the other hand, the FTs of intermolecular distances seen in Figure 6 look very similar to the AIMD results [38], indicating a clear change in vibrational frequency upon complexation of a metal cation.

**Figure 7:**
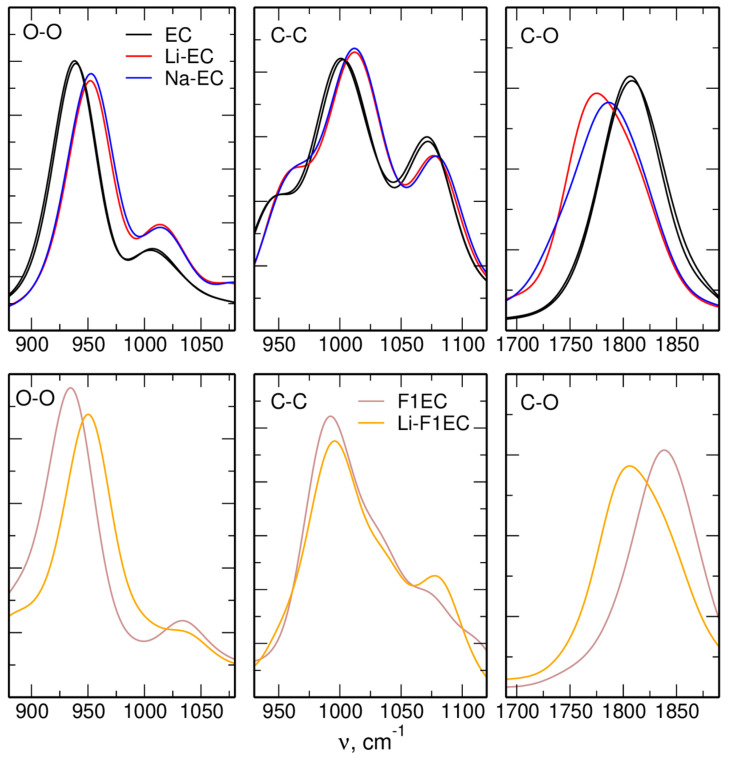
Averaged FTs of interatomic distances calculated from the GFN2-xTB simulations for MeTFSI electrolytes. Free and coordinated solvent molecules were averaged separately.

In order to compare the shifts calculated in the IR spectra to the shifts induced by ion–carbonate interactions, we followed the procedure used in [38], that is, we averaged the FTs over all solvent molecules within the sample, but separately for free and complexed molecules. The results are shown in Figure 7 for FTs of the C-O, O-O, and C-C distances. Note that for EC-based electrolytes, two lines of free carbonate molecules are shown, corresponding to the LiTFSI and NaTFSI solutions.

In the case of the C=O stretch, the shifts in the average frequency between free and interacting molecules are −32 cm^−1^ and −21 cm^−1^ for interactions with Li and Na ions, respectively. These values are 4–6 cm^−1^ larger than the shifts in the IR spectra, but there is an overall agreement between these two measures (as was also observed in the AIMD simulations). The shifts in the FTs of the O-O distance with a maximum at about 950 cm^−1^ are in the opposite direction and amount to 14–15 cm^−1^, regardless of the cation or the solvent. Qualitatively, this result is in agreement with the experimental data and AIMD results: interaction hardens the ring-breathing mode, but the shifts are smaller than those observed for the oscillations of the carbonyl group. Also, the FTs of the C-C distances exhibit a blue-shift, as was obtained from the AIMD data [38]. The main difference to the experiment and to the earlier simulations is the insensitivity of the shift to the cation type: in the xTB simulations, the shifts for both ions are the same, whereas smaller values were expected for the Na-EC electrolyte.

Therefore, we arrived at a question: why in the xTB data are the shifts of local oscillations of the ring inconsistent with the shifts in the calculated IR spectra (and the experiments), whereas agreement was found in the AIMD data? In trying to answer this, we should remember that the plots of the FTs in Figure 5, Figure 6 and Figure 7 show only the distribution of vibrational modes, not their IR intensity. To compute the latter, knowledge of the dipole moment is necessary; the oscillation has to change the dipole moment to carry the IR intensity. In AIMD simulations, the total dipole moment of the whole system was calculated at each step based on the electron density. In the xTB (and DFTB) simulations, the total dipole moment was calculated from the partial charges assigned to the atoms. A possible explanation for the disagreement between the FTs and IR spectra is that the charges fitted in xTB failed to correctly describe the dipole moment of the molecule coordinated to a cation. Better results were obtained for the C=O mode because it is well separated from other vibrational modes; the oscillating group interacts directly with the cation, and the oscillation leads to a significant change in the dipole moment. On the other hand, the ring-breathing vibration and the C-C oscillations are close in frequency to other modes, the change in the dipole moment is small, and the oscillation affects other parts of the molecule, not the interaction site directly. Therefore, the calculated spectrum in the range 900–1300 cm^−1^ can be sensitive to the balance of several factors, and its worse reproduction is hardly surprising.

To conclude, we should underline that the GFN2-xTB method allowed us to perform MD simulations for Li-containing electrolytes, which is a clear advantage over the DFTB approach, lacking the necessary parameters in readily available parameterizations. For these systems, the calculated structural data are comparable to AIMD results; a larger difference was observed for the solvation of Na^+^. A reasonable reproduction of IR spectra was obtained for neat solvents and also for interaction-induced shifts of the C=O stretching vibration. In the region of ethylene ring vibrations, the quality of the results is apparently worse. The GFN2-xTB MD simulations required computational effort about 1–2 order of magnitude lower than that required for the AIMD simulations in [38].

Discussing the possible ways of improving the semi-empirical results with respect to AIMD, we should note that the general parameterization of GFN2-xTB, beneficial for easy application of the method, becomes a disadvantage when a modification of parameters is considered. In fact, the GFN2-xTB method, like many other semi-empirical approaches, must be used “as is”. Tailoring of its parameters to a specific system would be against the philosophy behind the parameterization: of having only one, general-purpose parameter set. On the other hand, the DFTB approach with pair-specific parameter sets supplied to the software is particularly suitable for the development of users’ own parameterizations, designed for specific purposes. For the systems studied in this work, such a parameter set could improve the Me–carbonate and Me–anion interactions and possibly also the potential for atom pairs of carbonate molecules, thus yielding a better description of ring vibrations. The development of new DFTB parameterization requires, however, fitting the parameters using some reference data. Therefore, other possibilities of investing effort should be considered. One possible solution is the use of artificial intelligence methods to construct a machine-learned potential trained on the AIMD data, providing simulation accuracy close to that of DFT approaches but at a lower computational cost.

Nevertheless, the computational effort—significantly lower than that for DFT-based AIMD—still makes the GFN2-xTB approach an interesting alternative. It is, however, recommended to confirm its applicability for a system of interest by comparing the test results with experimental data and/or AIMD simulations.

## 4. Materials and Methods

The MD used in this study relies on the Born–Oppenheimer approximation, that is, the electron structure problem is solved at each step for given positions of nuclei. Based on the calculated potential energies and gradients, the classical Newton’s equations of motion yield the accelerations and velocities of atoms, which are then used to propagate the nucleus positions to the next step. The difference between the two approaches, denoted here as AIMD and GFN2-xTB, lies in the level of the QC method applied for electron structure calculations. In AIMD [38], we used the Kohn–Sham density functional theory methodology with the PBE functional [50], Goedecker’s pseudopotentials [51], and the molecularly optimized basis set of the DZVP quality. GFN2-xTB belongs to the semi-empirical methods, using empirical parameters to reduce the cost of calculations. It is a variant of the DFTB approach where the Kohn–Sham energy is expanded in terms of density fluctuations relative to a superposition of atomic reference densities. The key characteristics of GFN2-xTB are the following [48]. It uses a minimal valence basis set of atom-centered Gaussian functions. The GFN2-xTB Hamiltonian includes electrostatic interactions and exchange-correlation effects up to the second order in the multipole expansion. In the context of the present work, the most important feature is that the GFN2-xTB parameterization uses only global and element-specific parameters, and no pair-wise parameters are employed (as opposed to the DFTB approach).

**Figure 8:**
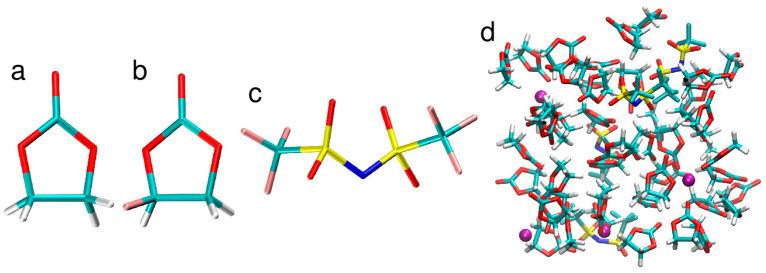
Structures of the EC molecule (**a**), F1EC molecule (**b**), and TFSI anion (**c**); a sample snapshot of the simulation box with 4 LiTFSI ion pairs in 46 EC molecules (**d**). Colors of atoms: C—cyan, H—white, O—red, S—yellow, N—blue, F—pink, Li—purple.

In this work, we studied the same systems as investigated by DFT/PBE AIMD simulations in our previous work [38], that is, neat EC and F1EC liquids, Li/NaTFSI solutions in EC, and LiTFSI solution in F1EC. Simulation boxes contained 50 carbonate molecules (neat solvents) or 4 Li/NaTFSI ion pairs in 46 solvent molecules. The molecules and anions studied in this work, as well as a sample snapshot of the simulation cell for LiTFSI/EC electrolyte, are shown in Figure 8. The composition of electrolytes corresponded roughly to a 1 M salt concentration. Three independent replicas were simulated for LiTFSI electrolytes, while two replicas were used for neat solvents and the NaTFSI/EC solutions. Initial structures of the systems were taken from the classical MD simulations and were the same as the starting points for the AIMD simulations in [38].

MD simulations applying the GFN2-xTB approach were performed in the DFTB+ v. 22.2 package [52]. The simulations were conducted for 45 ps in the NVT ensemble at T = 298 K with a time step of 1 fs. The Nosé–Hoover thermostat was used to control the temperature. The size of the periodic simulation cell was set to the value reproducing the density of the system obtained in classical MD simulations [38]. The last 40 ps of the trajectory was used for the analysis, and the results were averaged over the replicas of the system.

The IR spectra were calculated from the recorded trajectories as the Fourier transform of the autocorrelation function of the total dipole moment. To produce smooth plots, we convoluted individual peaks with Gaussian functions, setting σ = 5 cm^−1^. For the analysis of the local environments of solvent molecules, we calculated the FTs of three interatomic distances: the C-O distance in the carbonyl group and the C-C and O-O distances between the atoms from the ring of the carbonate molecule.

## Figures and Tables

**Table 1:** Positions of the maxima r_m_ in the Me–O_c_ RDFs (in Å) and the Me–O_c_ CNs obtained via AIMD and xTB simulations.

System	r_m_(Me–O_c_)		CN(Me–O_c_)	
	AIMD ^1^	GFN2-xTB	AIMD	GFN2-xTB
Li-EC	1.95	1.90	4.00	4.40
Li-F1EC	1.97	1.90	3.98	3.75
Na-EC	2.39	2.09	5.00	5.65

^1^ AIMD data from [38].

**Table 2:** Positions of the maxima r_m_ in the Me–O_T_ RDFs (in Å) and the Me–O_T_ CNs obtained in AIMD and xTB simulations.

System	r_m_(Me–O_T_)		CN(Me–O_T_)	
	AIMD ^1^	GFN2-xTB	AIMD	GFN2-xTB
Li-EC	-	1.78	0.0	0.28
Li-F1EC	-	1.78	0.0	0.71
Na-EC	2.4	1.99	0.2	0.85

^1^ AIMD data from [38].

**Table 3:** Shifts in the C=O stretching modes or ring-breathing vibrations in the calculated IR spectra (Δ_IR_), obtained from averaged power spectra (Δ_FT_) and the literature data (Δ_exp_). All values are in cm^−1^.

Vibration/System	Δ_IR_		Δ_FT_		Δ_exp_
	AIMD ^1^	GFN2-xTB	AIMD ^1^	GFN2-xTB	
C=O/Li-EC	−33	−27	−35	−32	−30 ^2^
C=O/Li-F1EC	−25	−28	−33	−32	−30 ^2^, −36 ^3^
C=O/Na-EC	−16	−15	−21	−21	−20 ^3^
O-O/Li-EC	-	-	9	14	12 ^4^
O-O/Li-F1EC	17	-	15	15	17 ^4^
O-O/Na-EC	-	-	4	14	-

^1^ AIMD data from [38]. ^2^ Data for acyclic carbonates from [17]. ^3^ Data for acyclic carbonates from [24]. ^4^ Data for cyclic carbonates from [17].

## Data Availability

Raw data are available upon request to the corresponding author.

## Sample Availability

Not available.
